# An Arch-Shaped Intraoral Tongue Drive System with Built-in Tongue-Computer Interfacing SoC

**DOI:** 10.3390/s141121565

**Published:** 2014-11-14

**Authors:** Hangue Park, Maysam Ghovanloo

**Affiliations:** GT-Bionics Lab, Georgia Institute of Technology, 85 Fifth St. NW, Atlanta, GA 30308, USA; E-Mail: hpark90@gatech.edu

**Keywords:** Intraoral Tongue Drive System (iTDS), assistive technology, brain-tongue-computer interface, system-on-a-chip, magnetic sensors, buccal shelf

## Abstract

We present a new arch-shaped intraoral Tongue Drive System (iTDS) designed to occupy the buccal shelf in the user's mouth. The new arch-shaped iTDS, which will be referred to as the iTDS-2, incorporates a system-on-a-chip (SoC) that amplifies and digitizes the raw magnetic sensor data and sends it wirelessly to an external TDS universal interface (TDS-UI) via an inductive coil or a planar inverted-F antenna. A built-in transmitter (Tx) employs a dual-band radio that operates at either 27 MHz or 432 MHz band, according to the wireless link quality. A built-in super-regenerative receiver (SR-Rx) monitors the wireless link quality and switches the band if the link quality is below a predetermined threshold. An accompanying ultra-low power FPGA generates data packets for the Tx and handles digital control functions. The custom-designed TDS-UI receives raw magnetic sensor data from the iTDS-2, recognizes the intended user commands by the sensor signal processing (SSP) algorithm running in a smartphone, and delivers the classified commands to the target devices, such as a personal computer or a powered wheelchair. We evaluated the iTDS-2 prototype using center-out and maze navigation tasks on two human subjects, which proved its functionality. The subjects' performance with the iTDS-2 was improved by 22% over its predecessor, reported in our earlier publication.

## 1. Introduction

Damage to the central nervous system that result in paralysis of the upper limbs. For instance, high level spinal cord injury and certain types of stroke, or double-amputation of the upper limbs, can lead to major difficulties in daily living of a considerable number of people due to loss of function, control, and independence [1]. Although caregivers can provide assistance to overcome some of these barriers, personal care is costly and often available only for a limited period of time. Individuals with severe disabilities desire more independence in their daily activities for tasks such as controlling a computer or driving a wheelchair [1]. To improve the quality of life for this group of people, researchers have developed many assistive technologies (ATs) that allow users to utilize their remaining abilities, such as control of the diaphragm, head, eyes, tongue, voice, and brain signals [2–8].

The tongue is well suited to control an AT because it is composed of strong and dexterous muscle fibers that do not easily fatigue as long as they are not required to apply force [9]. Also, the tongue is directly connected to the brain through cranial nerves, which are not affected by a spinal cord injury [10]. Furthermore, tongue-operated ATs protect the users' privacy because the tongue movements can be completely hidden from sight [11]. Several tongue-operated ATs have been developed to utilize the power of the tongue, such as the tongue-touch keypad (TTK), optical tongue sensing retainer, joystick-based tongue-point, tongue mouse using pressure sensors, inductive tongue control system (ITCS), and the Tongue Drive System (TDS) [12–17]. Every one of these tongue-operated ATs has its pros and cons in terms of functionality, usability, and acceptability among its potential users. For example, TTK, tongue-point, and tongue mouse require physical force from the tongue, which may cause fatigue on the tongue, and ITCS and TDS require users to receive a tongue piercing or attach a tracer on their tongue, which may cause temporary discomfort [18]. Nonetheless, researchers are still searching for better solutions to utilize the capabilities of the tongue while improving the user's comfort.

The basis for the TDS operation is monitoring changes in magnetic field, generated by a small magnetic tracer attached near the tip of the tongue, using an array of magnetic sensors distributed inside or around the oral space, and processing the resulting data to track the voluntary tongue position in real time [19]. The magnetic field generated by the tracer is not affected by the human body, and despite other sources of magnetic interference, such as the earth's magnetic field, offers the necessary accuracy to track the tongue position. We have developed signal processing algorithms that improve the signal to noise ratio (SNR) and recognize user-defined patterns of magnetic sensor signals that correspond to the tongue commands [20–23].

A key advantage of the TDS over the other tongue-operated ATs [13–17] is its ability to detect the tongue position in the 3-D intraoral space, which allows users to define a virtually unlimited number of commands by moving their tongues to various desired locations, as long as they can remember and repeat those tongue gestures. In addition, the TDS does not need any steering force or pressure applied by the tongue, which can result in tongue fatigue in several other tongue-operated ATs [13,15,16,18]. The TDS was first developed in the form of a headset, referred to as the external TDS (eTDS), with a pair of poles locating the magnetic sensors near the cheeks to measure the tracer magnetic field with sufficient SNR. The eTDS headset has been evaluated and its performance has been compared with a popular AT, called Sip’n’Puff, through two rounds of clinical trials in two rehabilitation centers [24–26].

**Figure 1.**
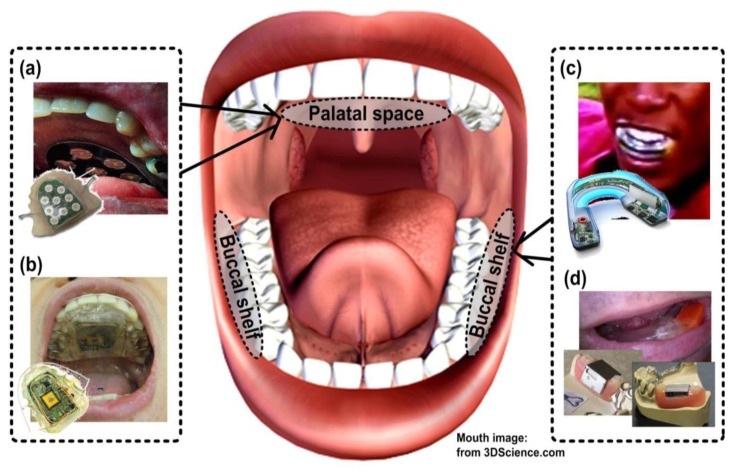
Intraoral appliances with built-in electronics: (**a**) Tongue-Touch Keypad; (**b**) iTDS-1; (**c**) X2impact mouth-guard and (**d**) Intellidrug intraoral drug-delivery system.

To improve the mechanical stability of the system and better protect the users' privacy, the intraoral version of the TDS (iTDS) is designed to locate inside the mouth on a dental retainer that clasps onto the teeth [12,27,28]. The first prototype of the iTDS, referred to as the iTDS-1 in this paper, was implemented in the form of a palatal retainer [12]. Palatal dental retainers locate in the palatal vault space, between the tongue and the palate, a space naturally designated for tongue movements in speech and a temporary reservoir of food during ingestion [29]. Several smart intraoral devices have been implemented as palatal dental retainers, such as the TTK, the ITCS, and the oral electro-tactile display [12,13,17,30]. Figure 1a,b shows the actual implementation of the TTK and the iTDS-1, occupying the palatal vault space.

There are several drawbacks in locating the iTDS-1 in the palatal vault space. Firstly, the iTDS-1 limits the palatal space and impedes proper tongue posture and movement necessary for clear speech [29]. Secondly, limiting the palatal space potentially degrades the iTDS performance because that space is needed for spacing out the tongue command locations and facilitating their easy selection. Intraoral space is also needed for passage of the tongue from one command location to another rapidly and naturally. Therefore, any object on the way might hinder the natural tongue movement [12]. Finally, when the iTDS is worn on the palate, it blocks the palatal tactile feedback that helps users to acquire a better sense of the magnetic tracer position and reproduce accurate tongue commands.

To preserve the palatal vault space, a new arch-shaped iTDS, which is referred to as the iTDS-2, has been designed to be located on the lower jaw, filling the buccal shelf area. The buccal shelf area, a space between molar teeth and inner cheeks, works as a primary stress-bearing area of the mandibular arch and has been used for appliances, such as a mouth-piece or a denture [31]. Figure 1c,d show examples of smart intraoral appliances with built-in electronics, designed to be worn on the lower jaw, in the buccal shelf area. We have selected the lower jaw over the upper jaw, to minimize protrusion of the lips and preserve the intraoral space. The intraoral anatomy provides less space between the upper teeth and upper lip than that the lower teeth and lip [29]. Moreover, when the mouth is open, it is easier for the tongue to maintain its relative position with respect to the lower jaw than the upper jaw. This makes it easier to issue the iTDS commands. Nonetheless, a detailed comparative assessment of various intraoral spaces in terms of efficacy and comfort level for a wireless, non-contact tongue-based control surface is needed.

In addition to the shape change resulting in the aforementioned advantages, the iTDS-2 has a more robust wireless communication capability compared to the iTDS-1, which is essential for the ATs that users fully depend on for their activities of daily living. The dual-band transmitter (Tx) in the iTDS-2 has a higher output power to provide the required SNR at a designated operating distance. We have carefully designed an antenna for the 432 MHz band to maximize the efficiency of radiated power towards the receiver (Rx). To reduce the probability of malfunction caused by strong external interference, the iTDS-2 automatically switches between its two bands based on the wireless link quality. A built-in super regenerative receiver (SR-Rx) in the iTDS-2 not only detects the wireless link quality in the 27 MHz band but also communicates with the TDS universal interface (TDS-UI) to receive the wireless link quality of the 432 MHz band. Accordingly, the iTDS-2 can switch between the 27 MHz and 432 MHz bands without any physical intervention by users.

To secure more than 24 h of operating time for the iTDS-2, *i.e.*, one full day of usage without any anxiety of running low on battery, we reduced the iTDS-2 power consumption by removing the microcontroller (MCU), which turned out to be the most power consuming block in the iTDS-1. Instead, we employed an ultra-low power field-programmable gate array (FPGA) and a built-in successive approximation register analog-to-digital converter (SAR-ADC), which replaced the MCU functions while consuming less power. Section 2 explains the iTDS-2 SoC architecture with circuit blocks for the wireless link and implementation of an arch-shaped dental retainer. Section 3 describes measurement results of each block and the overall system along with experiments done by two able-bodied participants for system evaluation, followed by concluding remarks in Section 4.

## 2. Design and Implementation

### 2.1. SoC Design

**Figure 2.**
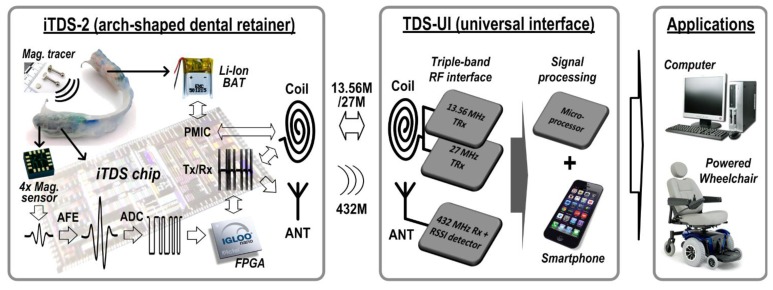
Overall system block diagram of the iTDS-2 including the TDS-UI and target devices.

Figure 2 shows the overall block diagram of the iTDS-2 with the TDS-UI and target applications. The iTDS-2 consists of four 3-axis magnetometers (HMC1043, Honeywell, Golden Valley, MN, USA) measuring the magnetic field generated by the magnetic tracer on the tongue, a FPGA generating digital control signals, and a system-on-a-chip (SoC) that amplifies, filters, digitizes, and wirelessly transmits the sensor data. The TDS-UI receives the raw magnetic sensor data from the iTDS-2, processes the data, and classifies it into user-defined commands that are delivered into target devices, such as a computer or a powered wheelchair.

**Figure 3.**
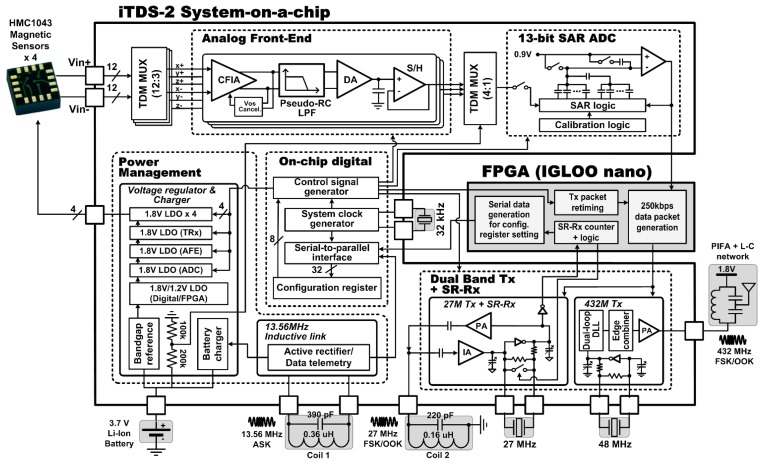
Detailed block diagram of the iTDS-2 system-on-a-chip (SoC).

Figure 3 shows the detailed block diagram of the iTDS-2 SoC that is architecturally similar to the iTDS-1 SoC in analog front-end (AFE), dual-band Tx, and power management (PM) blocks. The iTDS-2 also employs several new circuit blocks over the iTDS-1, such as a 13-bit SAR ADC and a SR-Rx.

The AFE in the iTDS-2 employs four gain steps (25, 50, 100, and 200). A current-feedback instrumentation amplifier (CFIA) is followed by a pseudo-RC low-pass filter and a differential amplifier, similar to [32]. The CFIA is equipped with offset cancellation, an auto-zeroing scheme, and a correction current that is applied accordingly to balance the CFIA until the next readout [32]. The 27 MHz carrier is generated by an internal oscillator circuit that has a 27 MHz off-chip ceramic crystal, and the 432 MHz carrier is generated by nine voltage-controlled delay cells in a dual-loop DLL that has a 48 MHz off-chip ceramic crystal. The class-C power amplifier generates on-off-keying (OOK) or frequency-shift-keying (FSK) outputs at 27 MHz, while the edge combiner operates as a nonlinear amplifier to generate OOK or FSK outputs at 432 MHz [12]. The PM block consists of voltage regulators, data telemetry, and battery charging circuits. The data telemetry block has bidirectional capability. Forward data telemetry consists of data and clock recovery circuits, while back telemetry consists of a load-shift-keying (LSK) mechanism built into an active rectifier [33]. Details of the AFE, Tx, and PM blocks can be found in [12].

While the iTDS-1 used the ADC built in the MCU, the iTDS-2 has an on-chip 13-bit SAR ADC to reduce power consumption and loading effect on the AFE output stage. The SAR ADC operates at 1024 Hz and employs noise shaping and foreground digital calibration schemes, to decrease the digitization noise by pushing it out into a higher frequency band and to minimize the effects of capacitor mismatch, respectively [34,35]. For noise shaping, the residual charge at the end of each conversion process is delivered to the next conversion stage, using the switched capacitor connected in parallel to the capacitor bank [34]. For calibration, the residual voltages are digitized by the ADC itself and the capacitor mismatches are calculated accordingly. The calibration results are stored in the microprocessor of the TDS-UI to correct the capacitor mismatch in advance [36].

**Figure 4.**
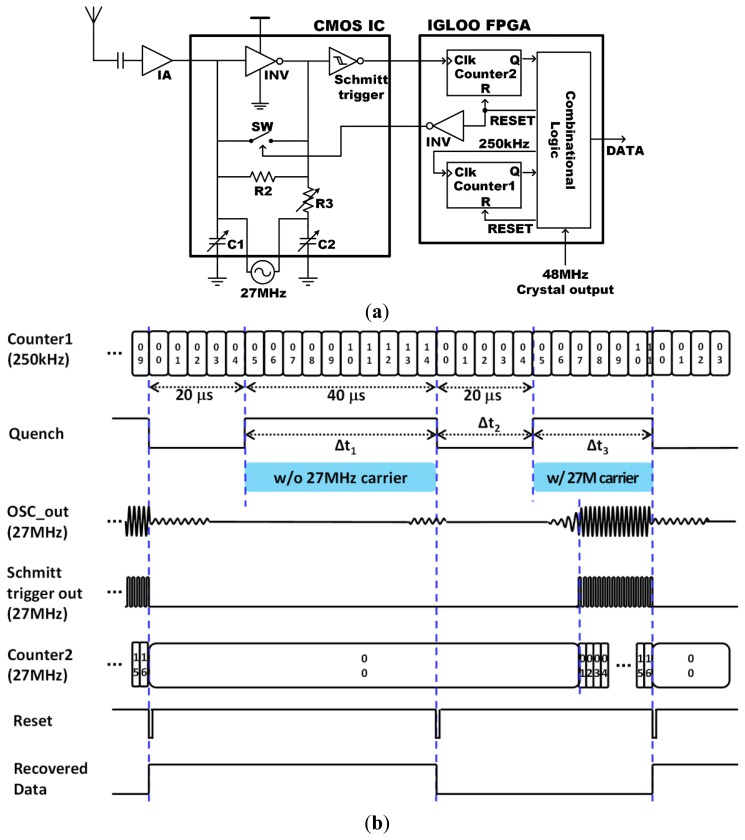
Digitally assisted SR-Rx: (**a**) block diagram and (**b**) timing diagram.

The SR-Rx was added to the iTDS-2 to overcome the limitation of the one directional communication in the iTDS-1, and to improve the system robustness. The SR-Rx is designed to detect the 27 MHz carrier and recover the OOK-modulated data up to 5 kbps. Figure 4a shows the SR-Rx implemented with an isolation amplifier, a 27 MHz quench-controlled oscillator, a Schmitt trigger, and part of the FPGA. Because of the power constraint in the iTDS-2, the SR-Rx incorporates a digitally-assisted self-quenching architecture, which reduces the power consumption by turning off the oscillation following each bit [37]. The FPGA counts the number of oscillations and turns off an oscillator faster than the conventional analog self-quenching architecture using a peak detector [38]. An additional advantage of the digitally-assisted self-quenching architecture is the reduced quench period, which can increase the maximum data rate.

Figure 4b shows how the SR-Rx works, with and without the 27 MHz RF carrier being present. Counter1 in the FPGA generates a 250 kHz real-time clock from the 48 MHz crystal output. The quench stays low for 20 μs to suppress the previous oscillation sufficiently, and stays high for 40 μs to detect the 27 MHz RF carrier. If an oscillation is detected within the 40 μs oscillation window, counter2 in the FPGA counts the number of oscillations and resets the quench after detecting 16 oscillations. The 40 μs oscillation window adjusts the sensitivity of the SR-Rx to −73 dBm to have 5 dB safe margin, considering the 10 dBm output power from the 27 MHz Tx in the TDS-UI and the 78 dB worst-case attenuation at the maximum operating distance of 22 cm [39]. Data is demodulated as ‘1’ or ‘0’ according to the counter1 value at the falling edge of the quench signal. Both counters are initialized at the falling edge of the quench signal to be ready to detect the presence of the 27 MHz RF carrier for the next bit. The resulting signal period is equal to or shorter than 60 μs, depending on the strength of the 27 MHz RF carrier. Considering that we adopted a 5 kbps data rate (200 μs period), the SR-Rx has an oversampling ratio of more than 3.3, which is sufficient to recover the OOK-modulated data [40].

### 2.2. Dual-Band Radio

To improve the robustness of the wireless link in the face of strong interference, the iTDS-2 incorporates a dual-band radio, which automatically switches the communication band between 27 MHz and 432 MHz. The wireless link quality is detected by the SR-Rx in the iTDS-2 and a CC1110 transceiver (Texas Instrument, Dallas, TX, USA) in the TDS-UI, for 27 MHz and 432 MHz, respectively. The SR-Rx detects the interference at 27 MHz when the strength of the interference is higher than −73 dBm, the sensitivity of the SR-Rx. The CC1110 detects interference at 432 MHz when the packet error rate (PER) is above 0.3%, which corresponds to the bit error rate (BER) of 10^−5^ for 296 bits in a packet [41]. When CC1110 detects the interference, the 27 MHz Tx in the TDS-UI sends an 8-bit Ack packet to the iTDS-2 for three times. When the SR-Rx in the iTDS-2 detects the 27 MHz carrier, the FPGA stores the incoming 8-bit data stream, continuously updates the data in a first-in first-out (FIFO) buffer, and compares it with a predefined Ack sequence (10100110) to determine whether the input is an Ack packet or a mere interference. If the FPGA recognizes an Ack packet, it changes the communication frequency from 432 MHz to 27 MHz, and vice versa if an Ack packet is not detected in the presence of 27 MHz interference.

**Figure 5.**
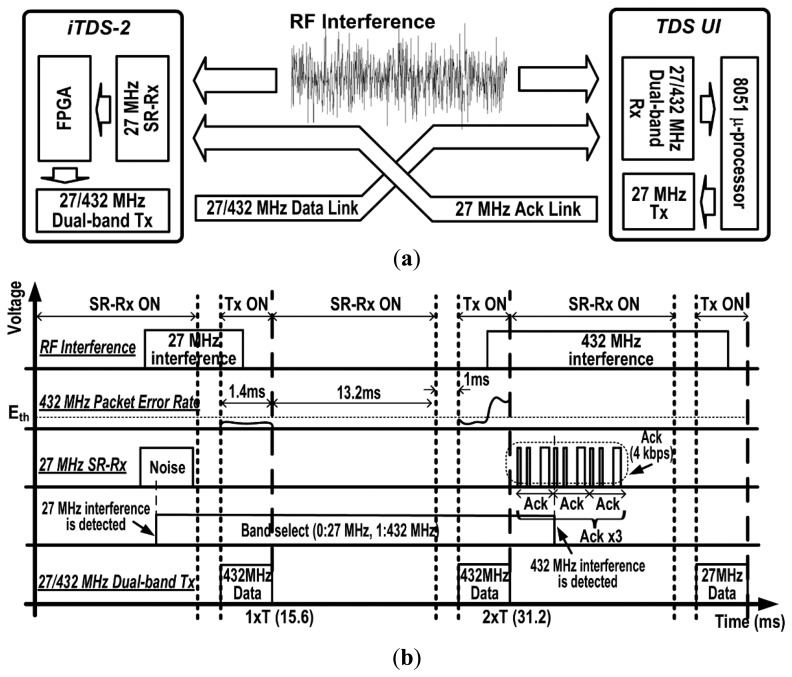
Dual-band data link operation at 27/432 MHz and the Ack link at 27 MHz: (**a**) block diagram and (**b**) timing diagram.

Figure 5 describes how the dual-band data link between the iTDS-2 and the TDS-UI works. For 13.2 ms time interval between data transmissions, the SR-Rx monitors interference at 27 MHz and receives information about interference at 432 MHz via the 27 MHz Ack link. The recovered data packet by the SR-Rx is fed into the FPGA to switch the communication band between 27 MHz and 432 MHz. The left side of the Figure 5b shows a transition from 27 MHz to 432 MHz and the right side shows a transition from 432 MHz to 27 MHz. The dual-band Tx transmits the magnetic sensor data within 1.4 ms via the selected communication band. Note that we assume at least one of the communication bands has a good wireless link quality. The dual-band Tx will continuously switch the band if both communication bands have poor wireless link quality below the threshold.

### 2.3. Antenna Design

For 432 MHz band, data is delivered by electromagnetic propagation at a nominal range of 0.3–2 m for environmental access and wheelchair navigation [12,42]. The antenna geometry should be carefully designed to optimize the wireless link propagating from the intraoral environment [43]. We selected a planar inverted-F antenna (PIFA) by considering three important parameters: radiation efficiency, antenna gain (directivity), and polarization [44,45]. Radiation efficiency can be increased by the PIFA because it has a wide radiation surface while it can be folded into a thin structure inside the arch-shaped dental retainer. The PIFA has limited backward radiation, which minimizes the electromagnetic power absorption towards the back of the head, while enhancing forward radiation towards the thinnest part of the intraoral surrounding to deliver more power to the TDS-UI receiver. Finally, the PIFA exhibits moderate to high gain in both vertical and horizontal states of polarization, which is also proper for the iTDS where the angle between Tx and Rx antennas changes according to the users' body posture [46–48].

**Figure 6.**
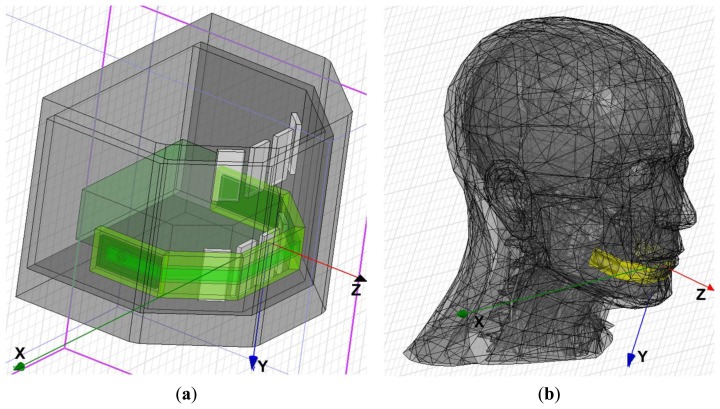

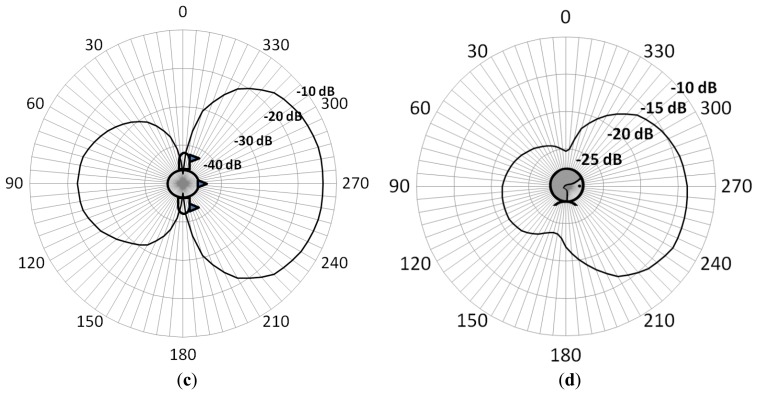
Simulation results of the PIFA: (**a**) a PIFA model inside the simplified mouth model; (**b**) a PIFA model inside the Ansoft-Aarkid human head model; (**c**) a radiation pattern at the horizontal plane at Φ = 270°; and (**d**) a radiation pattern at the vertical plane at θ = 270°.

The PIFA was simulated using HFSS software (ANSYS, Canonsburg, PA, USA). Figure 6a shows the PIFA model mounted onto a simplified human mouth model used for the initial tuning. Figure 6b shows the PIFA model mounted onto the Ansoft-Aarkid human head model used for the simulation results, for which we applied the actual frequency characteristics of tissues, bones, and materials of the iTDS-2. Figure 6b,c shows the horizontal and vertical radiation patterns (over *x*-*z* and *y*-*z* planes), respectively, which confirm the strong forward radiation characteristic of the PIFA. An average radiation efficiency of 9.26% was obtained from these simulations.

Impedance matching is another important factor in antenna performance to minimize the power loss caused by reflection from the antenna input back to the transmitter output [49,50]. In this design, the CLC network connected at the antenna feed-point, adjusts the antenna input impedance to be matched with the output impedance of the on-chip transmitter [51]. It is, however, difficult to avoid impedance mismatch because the mouth environment is continuously changing with the tongue and jaw motion, and the impedance of the antenna is changing accordingly. We plan to address this issue by incorporating an adaptive impedance matching scheme, which we have already tested in a prototype using a commercial-off-the-shelf (COTS) version of the iTDS [39].

### 2.4. System Implementation

**Figure 7.**
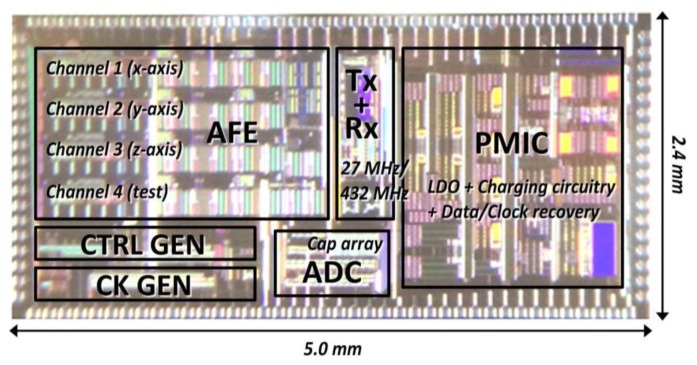
Die photo of the iTDS-2 system-on-a-chip, implemented in a 0.35-μm 2P3M std. CMOS process.

Figure 7 shows the iTDS-2 SoC, which has been implemented in a 0.35-μm 2P3M std. CMOS process, with the chip area of 5.0 × 2.4 mm^2^, including PADs.

**Figure 8.**
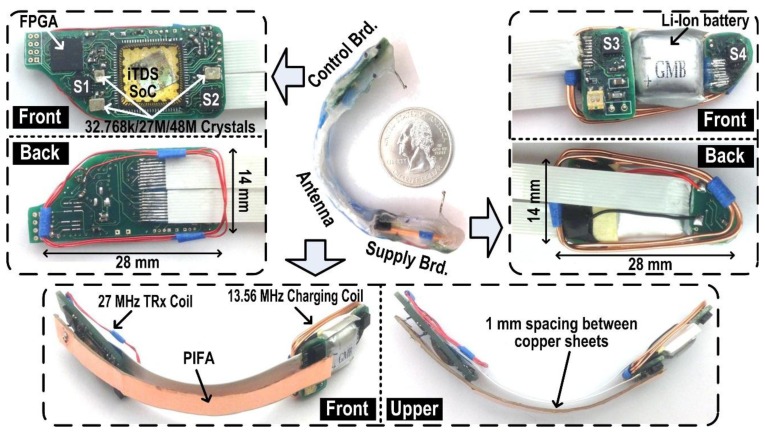
iTDS-2 prototypes with custom PCBs, electronics, a rechargeable Li-Ion battery, and a PIFA antenna, all hermetically sealed in the form of an arch-shaped dental retainer.

Figure 8 shows the iTDS-2 prototype, implemented as an arch-shaped dental retainer, to be mounted on the buccal shelf. The arch-shaped dental retainer is made of orthodontic Ortho-Jet crystal powder and liquid (Lang Dental, Wheeling, IL, USA) with two stainless still ball clasps (Patterson Dental Supply, St. Paul, MN, USA). The Ortho-Jet crystal covers the iTDS-2 electronics and protects them from external forces and the saliva, while the two ball clasps help fix the iTDS-2 onto the lower molar teeth. The iTDS-2 uses smaller ball clasps than usual (ϕ = 0.7 mm) because it utilizes inward clamping, while dental retainers such as Hawley retainer often utilize outward clamping.

To determine the maximum allowable size of the PCB and electronics, we reviewed literature on the buccal shelf area, which is defined as the space bounded on the medial side by the crest of the residual ridge, on the lateral side by the external oblique ridge, in the mesial area by the buccal frenulum, and on the distal side by the masseter muscle [31]. The length of the buccal shelf area is about the length of three consecutive molar teeth, reported as ∼30 mm, on average [52]. The height of the buccal shelf area can be defined as the total height of molar teeth including both the crown and root lengths. According to [53], the heights of the 1st, 2nd, and 3rd molars were 19.3 mm, 18.4 mm, and 17.2 mm, respectively, in 46 healthy individuals. We considered the data on the thickness of dentures to find information on the depth of the buccal shelf area, which are ∼3 mm over the 1st and 2nd molars and 6.3 mm over the 3rd molar [54]. However, we understand that the data derived from dentures can differ from the data from people with normal teeth, because the shape of the buccal shelf gradually transforms in the absence of the teeth. Another important consideration, which was not included in this study, is the difference between the oral anatomy and available oral space in men and women. Moreover, although these numbers helped us in design of the iTDS-2 prototype, we still need to customize the iTDS-2 for each potential user because everyone has a different oral anatomy.

The conclusion from this data for an average adult to wear the iTDS-2 with minimum discomfort was 28 mm × 14 mm × 2 mm in length, height, and thickness, respectively. Moreover, as shown in Figure 8, both control and supply boards on the right and left sides of the retainer, respectively, were tapered on the posterior side, as a trapezoids, considering the shape of the buccal shelf area [31]. The Ortho-Jet crystal layer over the electronics was made thin to minimize the retainer volume while it still provides the mechanical support. The arch-shaped bridge across two boards, which supports two 5.5 mm wide, 10-wire flat cable interconnects, was designed to be very close to the lower teeth to minimize protrusion towards the lower lips. The overall volume of the arch-shaped iTDS-2 dental retainer prototype was measured 12 mL, which is 40% smaller than the iTDS-1 palatal retainer (20 mL), reported in [12].

The control board, located on the right buccal shelf area, includes the iTDS-2 SoC, an IGLOO nano (Microsemi, Aliso Viejo, CA, USA) FPGA, three crystals, and two magnetometers. The supply board, located on the opposite side, includes a 50 mAh rechargeable Li-Ion battery (12 × 15 × 5 mm^3^) and two magnetometers. Moreover, a 3-turn, 30-gauge, enamel-coated, magnetic wire encompasses the control board for 27 MHz RF link and a 3-turns, 22-gauge, enamel-coated, magnetic wire encompasses the supply board for the inductive data link and the battery charging at 13.56 MHz.

The PIFA is located on the front side of the bridge to transmit the data carrier at 432 MHz towards the front of the mouth. It is made up of two copper sheets, 12.7 mm wide and 0.05 mm thick, one connected to the 432 MHz Tx output and the other to ground with 2 mm 30-gauge copper wires. A 1 mm thick piece of rubber was placed between the two copper sheets to maintain the spacing between them.

## 3. Measurement Results of System Evaluation

### 3.1. Measurement Results

We first checked if the SoC blocks operate well in terms of amplification, digitization, and wireless transmission, by checking the operation of each block individually and together. We then verified if the automatic switching of the frequency band works well with the SR-Rx operation, when there is interference at either 27 MHz or 432 MHz band. Finally, we measured the power consumption of each functional block and compared it with the results of the iTDS-1.

**Figure 9.**
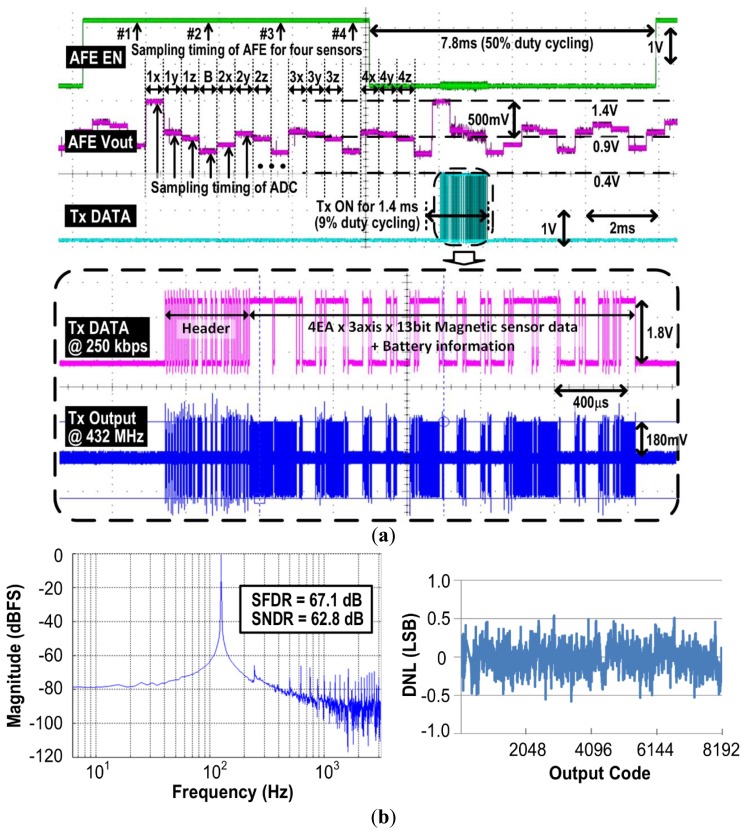
Measurement result of the AFE, Tx, and ADC: (**a**) a time domain waveform of the AFE and Tx within a single sampling period; (**b**) Output spectrum of a 128 Hz, 1.8 V_p-p_ sine wave input after fast Fourier transform and differential nonlinearity of the ADC over 10.1 bits ENoB.

Figure 9a shows measurement results of the AFE and the Tx, during a single sampling period acquiring data from four 3-axial magnetometers. Four 3-axial magnetometers generate 12 analog voltages that are digitized by the 13-bit SAR ADC and packetized by the FPGA. Following OOK/FSK modulation at 250 kbps, the Tx transmits the data wirelessly at either 27 MHz or 432 MHz. The data packet also includes battery voltage information to alert the user of the remaining charge. At 27 MHz, the data packet has start/stop bits for the universal asynchronous receiver/transmitter (UART) interface after recovery by the custom 27 MHz super-heterodyne receiver. At 432 MHz, the packet has a predefined 5-byte header to communicate with the CC1110 transceiver, as shown in Figure 9a [41]. The default output power at 27 MHz and 432 MHz is set to −3 dBm and −5 dBm, respectively.

The frequency spectrum in Figure 9b was measured at the output of the 13-bit SAR ADC with 128 Hz, 1.8 V peak-to-peak sine wave input, to evaluate the performance of the ADC, which results in 62.8 dB signal-to-noise-distortion ratio (SNDR) and 10.1 effective number of bits (ENOB). The differential nonlinearity (DNL) measurement result in Figure 9b shows that the DNL lies within ±0.54 LSB at 10.1 bits ENOB. The AFE and the ADC are turned on for half of the sampling period, while the high power blocks, such as magnetic sensors and the Tx are aggressively duty cycled at 2.3% and 9%, respectively, to save power and increase the operating time of the iTDS-2.

**Figure 10.**
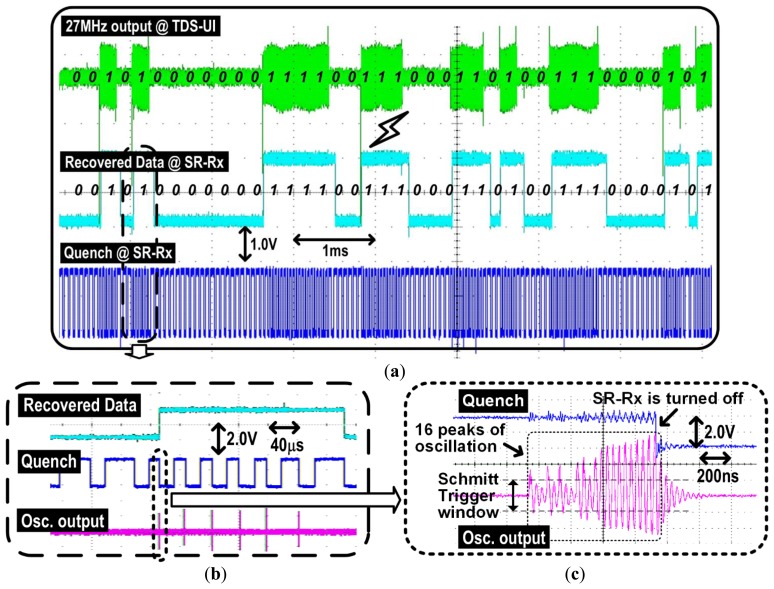
Measurement result of the digitally assisted SR-Rx: (**a**) an overall waveform with the transmitted/recovered data and the corresponding quench signal; (**b**) change of the quench period with the oscillation output according to the carrier; and (**c**) a magnified view of the oscillation output with the quench signal.

Figure 10a shows the measurement results of the SR-Rx, with the transmitted/recovered data and the corresponding quench signal. These results show that the SR-Rx successfully recovers the 27 MHz OOK carrier at 5 kbps. Figure 10b shows how the quench period changes according to the 27 MHz carrier amplitude. The quench signal is turned off after 40 μs if there is no oscillation or as soon as an oscillation is detected, and turned on again after 20 μs that was found as sufficient time to suppress the residual oscillation. Figure 10c shows that the oscillator is turned off after 16 peaks are detected by the FPGA. A Schmitt trigger is located between the oscillator and the FPGA to recognize only meaningful oscillations out of the noise floor. The FPGA recognizes the period of the quench signal and recovers the data accordingly. The data bit is recovered as ‘1’ when the quench period is less than 56 μs, to have a 4 μs margin, and as ‘0’ otherwise. When the strength of the 27 MHz RF carrier exceeds −73 dBm, which is the sensitivity of the SR-Rx, 16 oscillations occur in less than 56 μs and the counter resets the quench signal early enough to recover the data bit as ‘1’.

**Figure 11.**
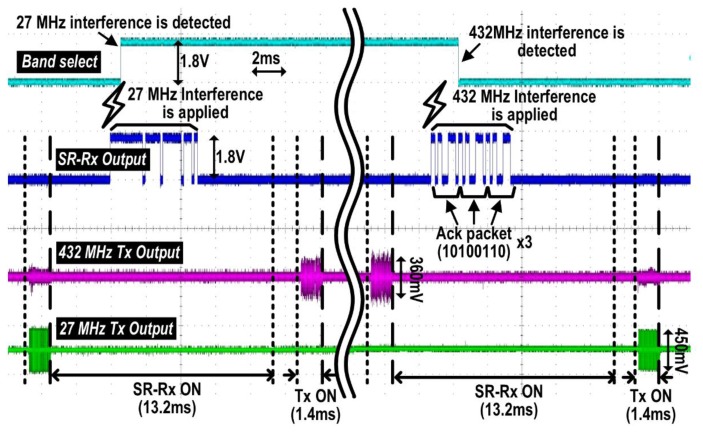
Operation of the dual-band radio when there is interference larger than the predefined threshold level at either 27 MHz or 432 MHz band.

Figure 11 demonstrates the operation of the dual-band radio when there is interference, larger than the predefined threshold level, at either 27 MHz or 432 MHz band. On the left side, the SR-Rx detects 27 MHz interference, and the Tx changes the communication band from 27 MHz to 432 MHz. On the right side, the TDS-UI detects 432 MHz interference by the built-in 432 MHz Rx and delivers the predefined 8-bit Ack packet to the iTDS-2 via 27 MHz band. The SR-Rx in the iTDS-2 detects the Ack packet and changes the communication band of the iTDS-2 from 432 MHz to 27 MHz accordingly.

**Figure 12.**
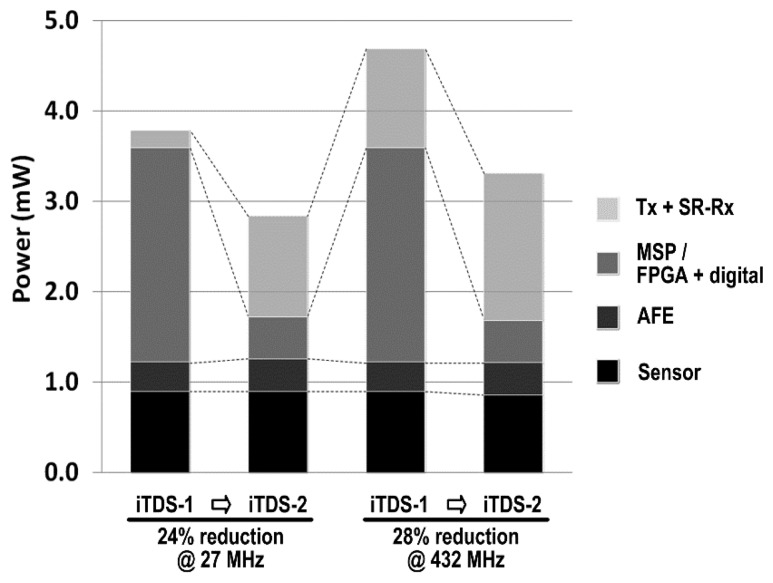
Comparison between the iTDS-1 and the iTDS-2 power consumptions.

Figure 12 compares power consumptions between the iTDS-1 and the iTDS-2. Compared to the iTDS-1, the average power consumption of the iTDS-2 is reduced by 24% and 28% to 2.8 mW and 3.3 mW in 27 MHz and 432 MHz bands, respectively. This is because of the built-in SAR-ADC and the low power FPGA in the iTDS-2, which have substituted an MCU (MSP430) in the iTDS-1, while consuming only 20% of its power. The Tx power consumption, however, is increased in the iTDS-2 to improve the wireless link robustness. Even though the Tx is turned on with only 9% duty cycle, its average power consumption is 1.12 mW and 1.62 mW, at 27 MHz and 432 MHz, respectively. The AFE and four magnetometers in the iTDS-2 consume 0.36 mW and 0.47 mW, with 50% and 2.3% duty cycling ratio, respectively.

### 3.2. System Performance Evaluation

**Figure 13.**
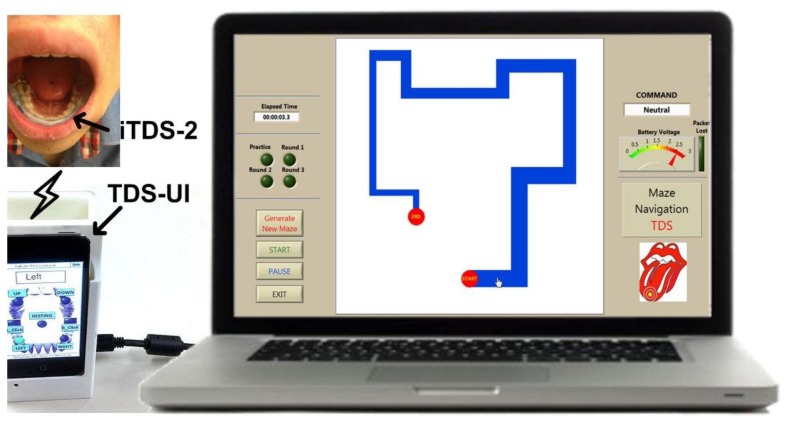
An experimental setup for the computer access test including the maze navigation GUI.

Figure 13 shows the experimental setup for the computer access test. After receiving approval from the institutional review board (IRB) at Georgia Institute of Technology, two healthy subjects (33-year old male and 30-year old female) participated in the experiment to evaluate the iTDS-2 performance. Subjects attached small disk-shaped magnetic tracers (Ø2.8 mm × 1.0 mm, K&J Magnetics, Jamison, PA, USA) on their tongues using dental adhesive and wore their customized iTDS-2 dental retainers on the lower jaw. The iTDS-2 transmits raw magnetic sensor data, changing with the tongue position, to the TDS-UI where the iPod-touch identifies one out of seven user-defined commands (left, right, up, down, left-click, right-click, and resting) and sends it to a computer via USB 55. Both subjects had already experienced the eTDS headset and the iTDS-1 palatal dental retainer on an occasional basis. However, this was their first time using the iTDS-2 arch-shaped dental retainer. Subjects trained the system with seven tongue commands and completed three trials for the maze navigation and center-out tapping tasks [55]. The graphical user interface (GUI) for the maze navigation task is shown in Figure 13.

**Figure 14.**
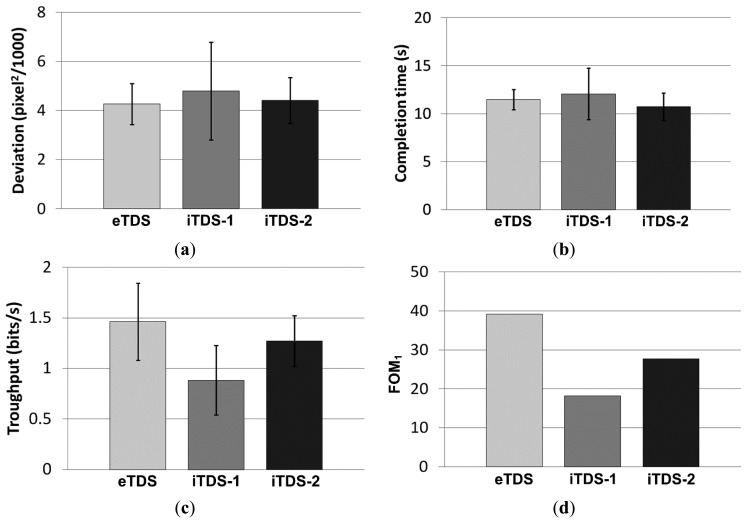
Performance comparison among the eTDS, the iTDS-v1, and the iTDS-2 with (**a**) the deviation at maze-navigation task; (**b**) the completion time at the maze-navigation task; (**c**) the throughput at the center-out task; and (**d**) FOM_1_ for six-command classification.

Figure 14 shows the performance comparison among the eTDS, iTDS-1, and iTDS-2. Figure 14a,b shows the deviation and completion time in the maze navigation task, respectively, and Figure 14c shows the throughput in the center-out tapping task. Deviation was calculated as the sum of area between the desired path on the maze and the actual traversed path of the mouse cursor on the screen, and completion time is the time taken by the subject to reach the end point. The throughput, measured in bits/s, is an indicator of the rate of information transfer from users to computers via a computer input device. Details of these tasks and their performance measures can be found in [55].

Subjects on average demonstrated 22% better performance with the iTDS-2 over the iTDS-1. We speculate that this is because the iTDS-2 does not limit the intraoral space, where the tongue moves, and also preserves the tactile feedback through the palate. The subjects' performance with the iTDS-2 is still inferior to that of the eTDS by ∼8% on average. The *FOM_1_* in Figure 14d indicates the quality of commands classification, which is found to be closely related to the subjects' performance in computer access [56]. It can be seen that the iTDS-2 is better than the iTDS-1 but worse than the eTDS, which is in agreement with the other performance measures. We speculate that the iTDS-2 might still be too bulky for easy, comfortable, and long term fit in the buccal shelf area, which might have caused the subjects' lower lips to be stretched a bit, degrading their performance. We should point out that the learning effect might have affected the experimental results, because the same subjects participated in the trials with the eTDS, the iTDS-1, and the iTDS-2. Also, there is not enough statistical power with only two subjects participating in the trial. We intend to conduct more experiments with a larger population over extended periods before drawing a solid conclusion regarding the iTDS-2 performance.

Table 1 shows the summary of the iTDS-2 specifications and benchmarking against other intraoral tongue-computer interfaces. The table shows that the iTDS-2 has the longest operating time compared to other tongue-operated ATs. The iTDS-2 also has a robust wireless link, thanks to a dual-band Tx that can automatically switch in the presence of interference in each band. Moreover, the iTDS-2 has a unique arch-shaped design to locate in the buccal shelf area, which preserves tactile feedback and more space for the voluntary tongue motion. We expect this feature to help the iTDS users with better spatial separation between their tongue commands, allowing for achieving a higher level of accuracy and increased number of tongue commands.

Table 2 summarizes the performance evaluation results of the iTDS-2 in the center-out tapping task and compares them with the iTDS-1, the eTDS, the ITCS, the sip-n-puff and a computer mouse using results from previous publications [12,25,57]. The iTDS-2 shows 73% improved performance over the sip-n-puff, which is one of the most popular ATs among those for tetraplegia. The computer mouse still outperforms the iTDS-2 by a factor of 3 to 1. However, this gap is expected to narrow as the iTDS users gain more experience and future versions of the iTDS become smaller, while using more power-efficient circuitry. Development of more advanced sensor signal processing algorithms to extract more information from the raw magnetic sensor data, while attenuating external interference and correcting user errors will also help with this trend.

## 4. Conclusions

We have presented a new version of the iTDS tongue-computer interface for individuals with severe physical disabilities. By being implemented as an arch-shaped dental retainer, the iTDS-2 preserves the intraoral space for tongue motion as well as the tactile feedback on the palate, both of which are expected to improve the system performance. The arch-shaped design also reduces the risk of interference with speech, which will be further evaluated in our future work. The iTDS-2 is equipped with a robust dual-band (27 MHz and 432 MHz) wireless link with increased output power compared to its predecessor (iTDS-1). A customized PIFA is fitted within the geometry of the iTDS-2 dental retainer and intraoral environment. It also monitors the link quality for each band in real time and automatically switches between them, if necessary. The built-in SAR-ADC and ultra-low power FPGA, which have replaced the MCU in the iTDS-1, increase the operating time of the iTDS-2 to ∼27.3 hours per charge of a small 50 mAh Li-Ion battery. The iTDS-2 performance was evaluated by two healthy volunteers wearing their customized iTDS-2 dental retainers. The experimental results showed that subjects achieved 22% better performance, on average, when they used the iTDS-2, compared to the iTDS-1. The results also show that the eTDS still outperforms the iTDS-2 by 8%. In future, we plan to implement the iTDS on a flex-PCB to further reduce the retainer thickness and volume, thus improving the user comfort. Moreover, we will add a real time adaptive matching circuit to the antenna feed point to compensate for dynamic variations in the oral space and further decrease the power loss caused by Tx-antenna impedance mismatch.

Like any other assistive technology or intraoral device medical that is meant for extended usage, safety is paramount in both functionality and performance of the iTDS, because it plays such an important role in the users' daily life, and it contains a rechargeable battery and other electronics. The current iTDS-2 prototype was developed only for acute experiments and coated in dental acrylic. Fortunately, hermetic sealing of the intraoral and implantable devices is well established. In the future versions of the iTDS, it will be coated with biocompatible polymers, such as Parylene-C, before being vacuum-molded in acrylic or thermoplastic. Additional safety and accelerated lifetime measurement tests are also necessary on the strength and the durability of the iTDS package and mechanical structure to avoid the possibility of crack formation or fragmentation under intraoral stress and aging.

## Figures and Tables

**Table 1.** Summary of specifications of the iTDS-2 and benchmarking with other intraoral tongue-operated ATs [12,14,17].

**Specifications**		**iTDS-2**	**iTDS-1 [12]**	**ITCS [17]**	**Optical Tongue Gesture [14]**
**Process**		0.35-μm std. CMOS	0.5-μm std. CMOS	Off-the-shelf	Off-the-shelf
**Die Area**		2.4 × 5.0 (mm^2^)	3.8 × 3.7 (mm^2^)	-	-
**VDD**		1.8 (V)	1.8 (V)	-	-
**Sensor**	**Type**	Magnetoresistive	Magnetoresistive	Inductive	Infrared proximity
	**Channel**	12	12	18	4
	**Sensitivity**	1.8 (mV/gauss)	1.8 (mV/gauss)	-	0.2–0.5 (V/mm)
	**Sampling**	64 (Hz)	64 (Hz)	30 (Hz)	90 (Hz)
**RF**	**Frequency Band**	27/432 (MHz)	27/432 (MHz)	2.4 (GHz)	Hardwired
	**Switching between Bands**	Automatic	Manual	Single band	
	**Data rate**	250 (kbps)	64 (kbps)	-	
**Power**	**P_avr_ (27 MHz)**	2.8 (mW)	3.7 (mW)	-	Hardwired
	**P_avr_ (432 MHz)**	3.3 (mW)	4.7 (mW)		
	**Battery Type**	Li-Ion 50 mAh	Li-Ion 45 mAh	Li-Ion 20 mAh	
	**Battery Lifetime**	27.3 (h), worst case	17.2 (h), worst case	15 (h)	
**Prototype**	**Shape**	Arch shape	Palatal shape	Palatal shape	Palatal shape
	**Size**	54 × 28 × 15 (mm^3^)	49 × 42 × 15 (mm^3^)	35 × 25 × 15 (mm^3^) *	41 × 38 × 15 (mm^3^) *
	**Volume**	12 (mL)	20 (mL)	-	-
	**Weight**	30 (g)	75 (g)	-	-

* Estimated from publication.

**Table 2.** Summary of performance of the iTDS-2 for a computer access task and comparison with iTDS-1, eTDS, ITCS, sip-n-puff, and mouse [12,25,57].

**Specifications**	**iTDS-2**	**iTDS-1 [12]**	**eTDS [25]**	**ITCS [57]**	**Sip’n’Puff [25]**	**Mouse [25]**
Throughput (bits/s)	1.25	0.83	1.48	0.85	0.72	3.66
Number of commands	6	6	6	8	4	-
